# Predictive Accuracy of Amyloid Imaging for Progression From Mild Cognitive Impairment to Alzheimer Disease With Different Lengths of Follow-up: A Meta-analysis

**DOI:** 10.1097/MD.0000000000000150

**Published:** 2014-12-12

**Authors:** Yan Ma, Shuo Zhang, Jing Li, Dong-Ming Zheng, Yang Guo, Juan Feng, Wei-Dong Ren

**Affiliations:** From the Department of Ultrasound (YM, JL, WDR), Department of Neurology (SZ, DMZ, YG, JF), Shengjing Hospital of China Medical University, Shenyang, Liaoning, China.

## Abstract

In the past decade, amyloid deposition has been shown to begin many years before the clinical symptoms of dementia in mild cognitive impairment (MCI) due to Alzheimer disease (AD). Longitudinal studies with different follow-up durations have suggested that ^11^C-Pittsburgh compound B positron emission tomography (^11^C-PIB-PET) may play a role in stratifying patients with MCI into risk levels for developing AD. However, the predictive accuracy of amyloid imaging for the progression from MCI to AD with different follow-up durations has not yet been systematically evaluated. A formal systematic evaluation of the sensitivity, specificity, and other properties of ^11^C-PIB-PET was performed.

This study aimed to systematically review and meta-analyze published data on the diagnostic performance of ^11^C-PIB-PET for predicting conversion to AD in patients with MCI and to determine whether long-term follow-up has a positive effect on predictive accuracy.

Relevant studies were systematically identified through electronic searches, which were performed in MEDLINE (OvidSP), EMBASE (OvidSP), BIOSIS Previews (ISI Web of Knowledge), Science Citation Index (ISI Web of Knowledge), PsycINFO (Ovid SP), and LILACS (Bireme). The methodological quality of each study was assessed by QUADAS-2. Sensitivities and specificities of ^11^C-PIB-PET in individual studies were calculated, and the studies underwent meta-analysis with a random-effects model. A summary receiver-operating characteristic curve (SROC) was constructed with the Moses-Shapiro-Littenberg method. Pooled estimates of sensitivity, specificity, positive likelihood ratio (LR+), negative likelihood ratio (LR−), diagnostic odds ratio (DOR), and the SROC curve of each subgroup were determined. Heterogeneity was tested, and potential sources for heterogeneity were explored by assessing whether certain covariates significantly influenced the relative DOR.

Eleven eligible studies consisting of a total of 352 patients with MCI at baseline were included. Overall, the studies were of moderate-to-high methodological quality. The sensitivity and specificity of ^11^C-PIB-PET for predicting conversion to AD ranged from 83.3% to 100% and 41.1% to 100%, respectively, with pooled estimates of 94.7% (95% confidence interval [CI]: 89.8%–97.7%) and 57.2% (95% CI: 50.1%–64.2%), respectively. Moderate heterogeneity was observed for specificity between the included studies (*I*^2^ = 42.1%). The pooled estimates for the long-term follow-up subgroup were 95.5% sensitivity (95% CI: 84.5%–99.4%) and 72.4% specificity (95% CI: 59.1%–83.3.8%), whereas the pooled estimates for the short-term follow-up subgroup were 94.4% sensitivity (95% CI: 88.2%–97.9%) and 51.0% specificity (95% CI: 42.6%–59.5%). Homogeneity in each subgroup was significantly higher than that of the included studies, and most diagnostic indicators in the long-term follow-up subgroup were far superior to those in the short-term follow-up subgroup or the entire group.

Not all of the methodological quality scores of studies included in this systematic review were high.

Current evidence suggests that prolongation of the follow-up duration tended to yield greater accuracy of ^11^C-PIB-PET for predicting the progression from MCI to AD. In particular, the specificity, which reflects the exploratory nature of the use of amyloid imaging to identify the process of MCI to AD, was improved with a longer follow-up period.

## Abstract 2

Supplemental Digital Content is available in the text

## INTRODUCTION

Alzheimer disease (AD) is a progressive and irreversible neurodegenerative disorder clinically characterized by memory loss and cognitive decline that severely affect the activities of daily living. The pathology of AD is present in the majority of cases of dementia. As the dominant or sole pathology, it accounts for over 50% of dementia. In some countries, the burden of AD dementia appears to be increasing faster than is generally assumed by the international health community.^1^

An increased cerebral β-amyloid (Aβ) burden in the form of fibrillar plaques or soluble oligomers and neurofibrillary tangles (NFTs) in the brain has been suggested to be the primary cause of brain degenerative changes and progressive cognitive deterioration in AD.^2^ However, the strength of the correlation between cognitive impairment and the pathological features of AD varies both with age and with each pathological feature. A significant number of individuals without clinical evidence of AD exhibit imaging evidence of amyloid deposition.^3^

Before patients with AD exhibit typical clinical symptoms of dementia, they present with a stage known as mild cognitive impairment (MCI),^4,5^ in which the patient has a degree of cognitive impairment greater than expected for their age but is not impaired in function. MCI is a heterogeneous condition that has been defined using clinical criteria by Petersen (1999).^5^ More than a dozen different definitions have been used to describe cognitive impairment that exceeds normal impairment and is qualitatively different from normal aging. While remaining attentive to the widely differing prognostic implications of the differing terms, in this review, the term MCI will be used to collectively describe the 16 conditions included in Matthews et al's study (2008).^6,7^ Four outcomes are observed for those within an MCI population: progression to AD dementia, progression to another dementia, maintaining stable MCI, or recovery. The National Institute on Aging (NIA) and the Alzheimer Association (AA) have published research criteria for MCI due to AD that incorporate the use of biomarkers to assess the likelihood that the MCI syndrome is due to the underlying pathophysiology of AD.^8^

The disappointing clinical trial results regarding modifying therapies in patients with AD suggest that the treatment strategy should be targeted to the MCI stage.^9^ Although there is no generally accepted diagnostic criterion sufficiently specific for predicting who will rapidly convert to AD among patients with MCI prior to the onset of symptoms in clinical practice, it is notable that amyloid deposition starts many years before the clinical symptoms of dementia in MCI due to AD are evident.^10^ Molecular neuroimaging, such as amyloid imaging, is useful not only to diagnose different subtypes of dementia but also for the early detection of AD and other neurodegenerative diseases.^11,12^ Amyloid positron emission tomography (PET) tracers provide a quantitative in vivo measure of the insoluble cortical Aβ load. To date, the most studied and validated PET marker of Aβ aggregation is ^11^C-Pittsburgh compound B (^11^C-PIB), which shows a nanomolar affinity for the extracellular and intravascular fibrillar deposits of Aβ and a low affinity toward the amorphous amyloid deposits, soluble Aβ, and intracellular NFTs.^13^^11^C-PIB-PET imaging may be essential for testing an Aβ immunotherapy drug on MCI patients with evidence of brain Aβ pathology, and it appears to be useful in assessing the effects of pre-dementia phase preventive treatments for potential AD on cortical fibrillar Aβ load in vivo. Therefore, proper application of ^11^C-PIB-PET would be useful to predict the conversion of MCI, which may have many prospects in clinical practice.^14,15^

Some longitudinal studies with different follow-up durations have suggested that ^11^C-PIB-PET may play a role in stratifying patients with MCI into their risk of developing AD and that as longitudinal studies continue to accumulate data, ^11^C-PIB-PET may become useful for predicting future clinical conditions, such as the risk of transitioning to AD. However, these studies require further replication and analysis in a pooled meta-analysis.^16,17^ Given the variability in their results and methodological quality, it is challenging to draw definitive and confident conclusions from these longitudinal studies with different follow-up durations. Additionally, recent research has suggested that the follow-up period may play an important role in the diagnostic accuracy for progressing from MCI to AD.^18^ Therefore, the aims of this meta-analysis were to systematically review the diagnostic efficiency of amyloid PET imaging using ^11^C-PIB-PET to predict the conversion to AD in patients with MCI; to explore whether different lengths of follow-up (short-term and long-term) have an effect on the prognostic value in longitudinal studies; and to discuss the relationship between the length of follow-up and predictive accuracy.

## METHODS

### Search Methods for the Identification of Studies

Relevant studies were identified through electronic searches, which were performed in MEDLINE (OvidSP), EMBASE (OvidSP), BIOSIS Previews (ISI Web of Knowledge), Science Citation Index (ISI Web of Knowledge), PsycINFO (Ovid SP), and LILACS (Bireme), for eligible studies published from 1999 (criteria for the diagnosis of MCI was proposed by Petersen [1999)]) through February 2014. The search strategy was performed with a combination of terms for titles and abstracts: (PiB or PIB or Pittsburgh compound B OR ^11^C Pittsburgh or PiB-PET or ^11^C PiB-PET OR amyloid ligand or [^11^C]PiB) AND (positron emission tomography or PET) AND (amyloid imaging or beta-amyloid or amyloid or amyloid-β or amyloid deposition) AND (mild cognitive impairment or MCI) AND (Alzheimer’ disease or Alzheimer disease dementia). The search was limited to articles including human subjects. Reference lists of retrieved articles and any relevant systematic reviews were screened for additional studies. No language restriction was applied to the electronic searches.

### Criteria for Included Studies

Studies were included if they fulfilled the following criteria:

- The study was a longitudinal cohort study in which amyloid imaging results were obtained at baseline, the reference standard (clinical outcomes) result of MCI was identified at follow-up, and the study included at least 10 MCI participants.^19^
- MCI participants at baseline met the Petersen criteria or revised Petersen criteria^4,5,7,20,21^; furthermore, core clinical criteria for MCI, proposed by the NIA-AA workgroup, were also acceptable.^8^ These criteria include subjective complaints, a decline in memory objectively verified by neuropsychological testing in combination with a history from the patient, a decline in other cognitive domains, no or minimal impairment of activities of daily living, and not meeting the criteria for dementia. Therefore, the eligible participants underwent a number of tests, for example, neuropsychological tests for cognitive deficits and checklists for activities of daily living, prior to study entry. MCI participants did not use acetylcholinesterase inhibitors or other neuropsychopathic medications during follow-up.
- The diagnoses of AD met probable National Institute of Neurological and Communicative Disorders and Stroke and the Alzheimer's Disease and Related Disorders Association (NINCDS-ADRDA) criteria of AD or the Diagnostic and Statistical manual of Mental disorders (DSM) and International Classification of Diseases (ICD)'s definitions for AD.
- MCI participants could be grouped into those with ^11^C-PIB positivity, and those with ^11^C-PIB negativity using validated PET imaging at baseline. The definition of ^11^C-PIB positivity was that ^11^C-PIB ligand uptake exceeded a certain threshold in the PET imaging protocol and vice versa.
- Follow-up periods for all MCI participants were at least 12 months, and clinical outcomes of MCI participants could be obtained at the last follow-up.

## DATA SYNTHESIS AND ANALYSIS

### Selection of Studies

Two authors (ZDM and ZS) performed the first assessment of the search results to remove the obvious non-relevant studies. Two authors (MY and FJ) independently reviewed the remaining abstracts and titles identified by the database searches for potentially eligible studies. If one or both reviewers considered the study potentially eligible, the full manuscript was evaluated against the inclusion criteria by both reviewers. Discrepancies were resolved by consensus. When necessary, a third arbitrator resolved disagreements not able to be resolved through discussion.

The following information was extracted from each eligible study:

- Basic clinical and demographic details of participants at baseline including age, sex, education, Mini-Mental State Examination (MMSE) score, apolipoprotein E(APOE)-ε 4 carrier status, number of subjects, MCI clinical criteria, MCI subtypes, sources of referral, participant recruitment, and sampling procedures. If there were several baseline time points, only data from the initial time point were used.
- Protocols for ^11^C-PIB-PET imaging consisting of the ^11^C-PIB test administration method, including the time between ^11^C-PIB injection and PET acquisition, thresholds used to define positive and negative tests, ^11^C-PIB dose, measures of ^11^C-PIB amyloid retention, and image analysis of discriminating brain regions.
- Procedures for the conversion to AD, including the reference standard of AD dementia used in included studies, the duration of follow-up from the time that ^11^C-PIB-PET imaging was performed to defining AD dementia by reference standard (similar to the criteria above, if there were several follow-up intervals in 1 study, only data from the endpoint of the longest follow-up period with composite data were used), prevalence or proportion of the population developing AD, and cumulative and per-year conversion rate with severity, if described.

The data were extracted independently by 2 blinded review authors (ZS and MY) and included the number of patients with MCI that converted to AD, number of nonconverters, number of true-positives (TPs), false-positives (FPs), false-negatives (FN), true-negatives (TN), sensitivity, specificity, positive likelihood ratio (LR+) and negative likelihood ratio (LR−). TP was defined as the number of MCI patients who had ^11^C-PIB-positive image results determined by PET, and the subsequent conversion to AD was confirmed at follow-up. TN was defined as the number of MCI patients who had ^11^C-PIB-negative image results determined by PET, and MCI due to AD was not confirmed by the end of follow-up. FP was defined as the number of MCI patients with ^11^C-PIB-positive image results that did not progress to AD during the follow-up. FN was defined as the number of MCI patients with negative imaging results, but the conversion to AD was eventually confirmed.

The sensitivity and specificity of individual studies were extracted and calculated using 2 × 2 contingency tables. Sensitivity was defined as the proportion of MCI converters who received the correct ^11^C-PIB-positive diagnosis determined by PET (TP/[TP + FN]). Specificity was defined as the proportion of MCI nonconverters who received the correct ^11^C-PIB-negative diagnosis determined by PET (TN/[TN + FP]). LR+ was calculated as sensitivity/(1-specificity), and LR− was calculated as (1-sensitivity)/specificity. LR+ indicates how much to increase the probability of progression to AD if the ^11^C-PIB-PET test of an MCI patient is positive, and LR− indicated how much to decrease the probability of progression to AD if the ^11^C-PIB-PET test of an MCI patient is positive. If the above data were unavailable from original article, the data were calculated by extracting the relevant data.

When a study did not present all relevant data necessary for creating a 2 × 2 table, we contacted the authors directly to request further information. The numbers lost to follow-up were recorded for each included study. We also extracted the data necessary for assessing quality, as defined below.

The time interval over which progression from MCI to AD occurs is very important; therefore, all included studies were divided into several subgroups according to different follow-up periods, if necessary (please see Thresholds Effect and Investigations of Heterogeneity). Specifically, we chose 1 year as the minimum period of delay in verifying the diagnosis (ie, the time between the last assessment at which a diagnosis of MCI was made and the assessment at which the diagnosis of AD dementia was made). The analysis was segmented into separate follow-up mean periods for the delay in verification: 1 year to <2 years; 2 to <4 years; and >4 years. This segmentation was performed to explicitly compare short- and long-term follow-up intervals and determine whether a long-term follow-up interval is more beneficial for using ^11^C-PIB-PET to predict MCI to AD conversion.

### Assessment of Methodological Quality

The methodological quality of the included studies was assessed using the Revised Quality Assessment of Studies of Diagnostic Accuracy Included in Systematic Reviews (QUADAS)-2 tool,^22^ as recommended by the Cochrane Collaboration. QUADAS-2 evaluates the risk of bias and concerns regarding the applicability for patient selection, index tests, reference standards, and risk of bias in the domain of study flow/timing. All included articles were analyzed in terms of patients (newly diagnosed MCI), index test (^11^C-PIB-PET), reference standard (AD dementia), and flow and timing. Studies were scored as “L” for low risk of bias/low concerns regarding applicability, “H” for high risk/high concerns, and “U” for unclear items for each domain. All domains with at least 1 negative response were scored as H regarding applicability, whereas domains with no negative responses but at least 1 unsure response were scored as U. Domains with no negative and no unsure responses were scored as L. Two blinded independent raters (GY and LJ) performed the QUADAS-2 assessment. Any disagreements were resolved by consensus or arbitration. For each individual study, the final results of the quality assessment were tabulated.

### Statistical Analysis

#### Data Synthesis

For each study, we constructed a 2 × 2 contingency table consisting of TP, FP, FN, and TN results, in which all participants were classified as presenting with positive or negative ^11^C-PIB-PET at baseline and being cognitively progressive or stable during the follow-up interval. To calculate the log OR (log odds of the true-positive rate and log odds of the false-positive rate), we added 0.5 to each cell in any 2 × 2 table with a value of zero.

If no threshold effect was observed, the pooled indices of sensitivity and specificity with the corresponding 95% confidence intervals (CIs) were calculated using weighted averages according to the sample size of each study. The pooled estimates of LR+, LR−, and diagnostic odds ratio (DOR) were computed using the DerSimonian and Laird method based on a random-effects model.

Subsequently, exploratory analyses were conducted by plotting estimates of sensitivity and specificity from each study on forest plots, and the results of the individual studies were also displayed in receiver-operating characteristic (ROC) space. A weighted symmetric summary ROC curve (SROC) with a 95% CI was computed with the Moses-Shapiro-Littenberg method according to different situations, and the quantitative value of the area under the curve (AUC) ± standard error (SE) was also calculated. Furthermore, we also defined the maximum joint sensitivity and specificity as point Q^∗^ on a symmetric ROC curve, which is a global measure of test accuracy.

^11^C-PIB-PET imaging test accuracy was evaluated according to the target condition. Currently, there is a set of acknowledged thresholds for defining ^11^C-PIB-PET positivity for MCI due to AD and AD dementia, and therefore, the estimates of diagnostic accuracy reported in primary studies were likely to be based on data-driven threshold selection^23^ unless prespecified. For studies in which researchers did not report a criterion for positive ^11^C-PIB-PET imaging results, the optimum threshold value was used as the reference standard, which was calculated by constructing a ROC curve from the study.

#### Thresholds Effect and Investigations of Heterogeneity

To determine whether researchers may have used different thresholds to define positive and negative test results (either explicitly or implicitly), a Spearman correlation coefficient between the logit of sensitivity and logit of 1-specificity was calculated to assess the presence of a threshold effect. A strong positive correlation (Spearman ρ > 0.6) suggested the presence of a threshold effect.^24^ Heterogeneity between the results of individual studies was tested by the inconsistency index (I^2^), which describes the percentage of total variance due to heterogeneity rather than chance across different studies. A zero percentage index indicates no heterogeneity, whereas 25%, 50%, and 75% indicate low, moderate, and high heterogeneity, respectively. To determine whether a potential source of heterogeneity resulted from certain covariates (ie, mean age, sex, MMSE score, APOE ε4 status, referral centers, MCI subtypes, follow-up time, MMSE score, PET protocols, reference standard and methodological features), meta-regression analysis was performed on the condition sufficient studies with available data were available. We considered variates explanatory if their regression coefficients were statistically significant (*P* < 0.05). In addition, if any level of heterogeneity or a significant covariate was identified, further subgroup analyses were performed.

#### Sensitivity Analyses

To evaluate the robustness of the meta-analysis, a sensitivity analysis of each subgroup was performed by excluding studies individually starting from the minimum to the maximum follow-up period to assess the influence of an individual study on the DOR. We also investigated the effect of prespecification of threshold and amyloid positivity on diagnostic accuracy by performing sensitivity analyses. Consistent results indicated stronger evidence of an effect and of study generalizability.

Publication bias was not investigated due to current uncertainty regarding how performance bias operates in test accuracy studies and the interpretation of existing analytical tools, such as funnel plots.^25^ The above opinions were supported by Cochrane systematic reviews of diagnostic test accuracy.^26–29^

All statistical analyses were executed with Stata, version 11 (Stata, College Station, TX) and Meta-DiSc statistical software, version 1.4 (Unit of Clinical Biostatistics, Ramón y Cajal Hospital, Madrid, Spain).

## RESULTS

### Study Identification

The computer-aided search revealed 2621 articles from electronic databases (APPENDIX 1). After de-duplication and first-assessments of abstracts, 89 articles remained and were screened for eligibility for this meta-analysis. After the first review of the full-text article, 55 articles remained. In a second round of screening of full-text articles, 44 articles were excluded for following reasons: no extractable data for constructing 2 × 2 tables to calculate sensitivity and specificity (n = 4); participants exhibited other conditions in addition to MCI, which could not be differentiated at baseline (n = 7); reference standards were for not only AD dementia but also other types of dementia, such as frontotemporal dementia, dementia with Lewy bodies, and dementia that could not be differentiated (n = 4); no delayed verification study (n = 8); results from a combination of other diagnostic modalities, such as neuropsychological tests, that could not be differentiated (n = 4); multiple publications, that is, the same data overlapped with another eligible study consisting of a larger number of patients in the study (n = 4); the tracers used in PET imaging were not only ^11^C-PIB but other radiotracers (ie, ^18^F-FDG, ^11^C-BF-227, ^18^F-FDDNP, ^18^F-AV-45, ^18^F-GE067, and ^11^C-SB-13) that could not be differentiated (n = 3); no threshold was used (changes in ^11^C-PIB levels measured over time) (n = 10) (see the PRISMA flow diagram, APPENDIX 2).

Thus, 11 studies remained, with a total sample size of 378 participants with MCI at baseline.^18,30–39^ Of the 352 participants with analyzable data, 151 developed AD, and 9 developed non-AD-type dementia. The remaining 26 participants were reported to be lost to follow-up. The majority (n = 18) of participants were missing from a single study.^36^

In some studies,^30,32–35,39^ the participants with MCI fell into 2 subgroups according to several criteria, such as sources of subjects, follow-up findings (progressive or stable), and PET imaging assessments (positive or negative). To achieve consistent expression, some quantitative variables at baseline from both subgroups were expressed as the mean ± standard deviation (M ± SD). The weighted pooled M ± SD was calculated using statistical methods. Two methods were utilized to measure the amyloid retention in Jack et al’ study (2010)^33^: transforming cerebrospinal fluid (CSF) Aβ42 measures into calculated ^11^C-PIB measures and ^11^C-PIB-PET imaging. The study pooled measures of Aβ from either source, and the number of amyloid-positive subjects that progressed from MCI to AD in whom amyloid retention was measured by ^11^C-PIB-PET was not reported in that article. Although transforming CSF Aβ42 into calculated ^11^C-PIB was shown to be an effective alternative to measuring amyloid load,^40^ to avoid the potential risk of bias, the authors were contacted, and the raw data from subjects with MCI measured by ^11^C-PIB-PET imaging were available. In addition, although some usable data were also obtained for 2 studies^36,37^ by contacting the authors of the studies, screening the reference lists of these articles did not result in other potentially relevant articles. The study sizes were small and ranged from 10 to 68. Five studies reported a mean age of >70 years,^18,33,34,38,39^ and 5 articles reported a mean age <70 years.^30,31,35,37^ Only 1 study^32^ reported a range of ages.

All of the studies were published within the last 5 years (2009–2014). Most studies (7/11) were conducted in Europe: 2 were conducted in Asia and the Pacific, and 2 were conducted in the United States. Nine included studies applied the NINCDS-ADRDA criteria as a reference standard for AD dementia; Koivunen et al^34^ also used DSM-IV criteria. One study^36^ did not explicitly state what reference standard was used at follow-up. The duration of follow-up was reported as the mean and SD, median, minimum duration, maximum duration, or range. The length of follow-up varied substantially but was mostly within a range of 18 to 50 months. The longest follow-up period of participants with MCI was found in Kemppainen et al’ study (2014).^18^ Because a significant difference was observed in the durations of follow-up between the long- and short-term follow-up studies, these included studies were divided into 2 subgroups classified as long-term and short-term according to whether the length of follow-up was >2 years. Six studies^32–34,36,38,39^ were classified as short-term, and 5^18,30,31,35,37^ were classified as long-term. The demographic and patient characteristics of the included studies are summarized in Table 1 and Table 2.^18,32–39^

**TABLE 1:**
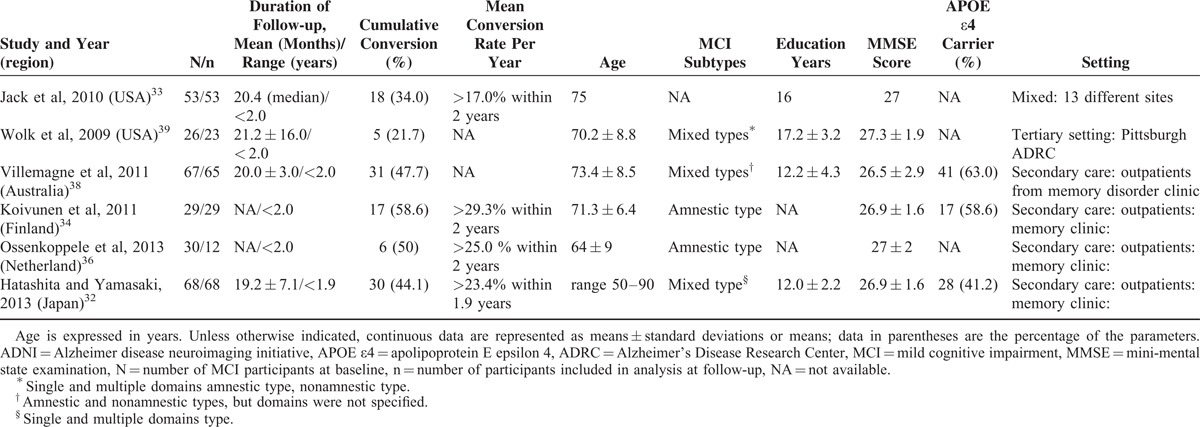
Demographic Characteristics of Participants with Mild Cognitive Impairment in Short-term Follow-up Subgroup

**TABLE 2:**
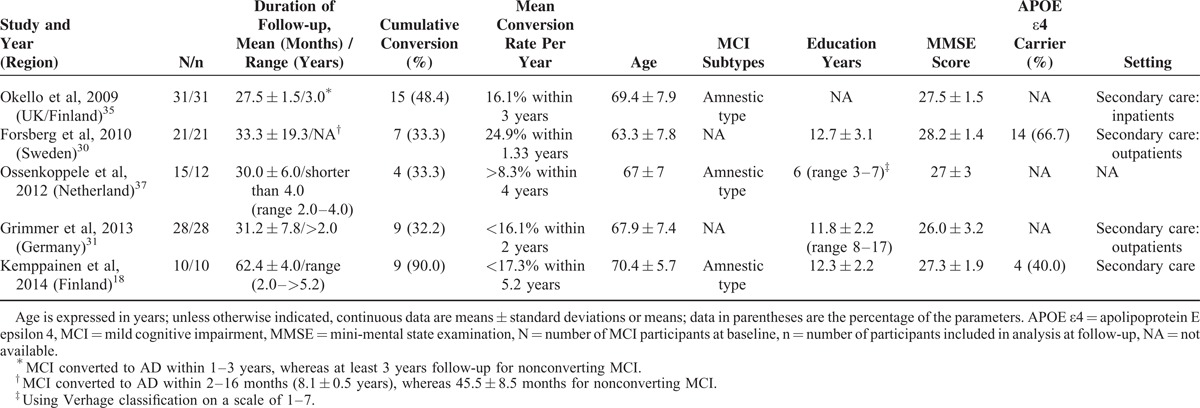
Demographic Characteristics of Participants with Mild Cognitive Impairment in Long-term Follow-up Subgroup

The included studies varied markedly in how the ^11^C-PIB-PET scans were performed and interpreted. Table 3 and Table 4 (APPENDIX 3, http://links.lww.com/MD/A79) summarize the data regarding the ^11^C-PIB-PET protocols.

### Methodological Quality Assessment

The review authors’ judgments regarding each methodological quality item for each included study are presented in Table 5 (APPENDIX 3, http://links.lww.com/MD/A79). The overall methodological quality of the studies according to the QUADAS-2 scores is displayed in Table 6 (APPENDIX 3, http://links.lww.com/MD/A79).

### Diagnostic Performance and Summary Estimates

#### Analysis of the Entire Group

**FIGURE 1:**
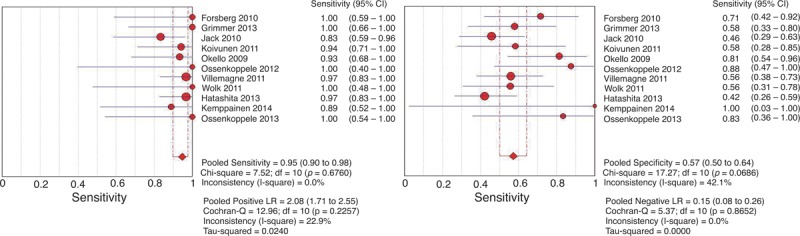
Forest plot of ^11^C-PIB-PET for predicting conversion to AD in patients with MCI. The figure shows the 2 × 2 table (TP, FP, FN, and TN) for each study, which form the basis for statistical analyses. Study-specific estimates of sensitivity and specificity are shown (represented as squares) with 95% CIs (represented as lines). Using Meta-DiSc, these estimates (and CIs) are also shown graphically. This figure demonstrates the greater uncertainty (indicated by CI width) and variability (indicated by the scatter of point estimates) in specificity compared with sensitivity. For the included studies, the sensitivities ranged from 83.3% to 100%, whereas the specificities ranged from 42.1% to 100%. The pooled sensitivity was 94.7% (95% CI: 89.8%–97.7%), and the pooled specificity was 57.2% (95% CI: 50.1%–64.2%). ^11^C-PIB-PET = 11C-Pittsburgh compound B positron emission tomography, 95% CI = 95% confidence interval, AD = Alzheimer disease, DOR = diagnostic odds ratio, FN = false-negative, FP = false-positive, MCI = mild cognitive impairment, TN = true-negative, TP = true-positive.

**FIGURE 2:**
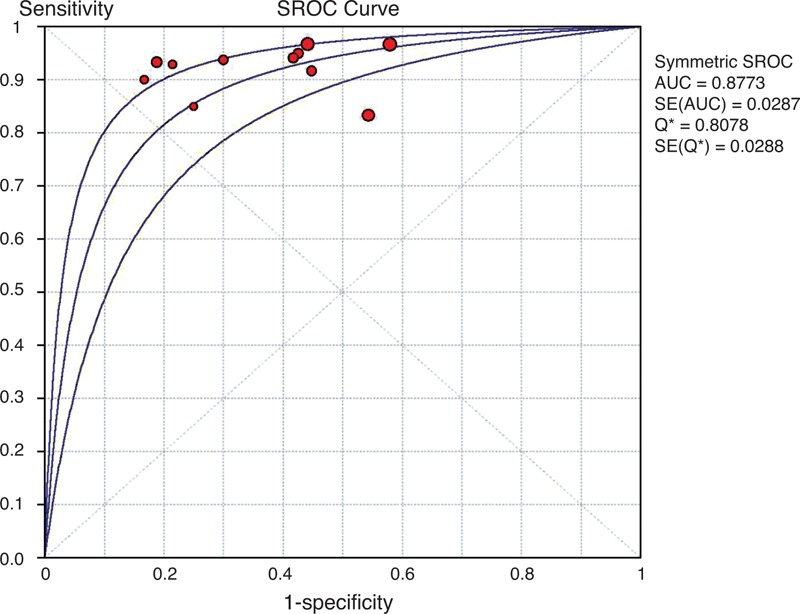
SROC curve of ^11^C-PIB-PET for predicting conversion to AD in patients with MCI. A weighted symmetric SROC curve with a 95% CI was computed with the Moses-Shapiro-Littenberg method, and the quantitative value of the AUC ± SE was 0.8773 ± 0.0287. We calculated the maximum joint sensitivity and specificity as point Q∗ on a symmetric ROC curve (0.8078 ± 0.0288), which is a global measure of test accuracy. Summary ROC plots display the results of individual studies in ROC space; each study is plotted as a single sensitivity–specificity circular point. The size of points in the plot is proportional to their sample sizes. ^11^C-PIB-PET = ^11^C-Pittsburgh compound B positron emission tomography, 95% CI = 95% confidence interval, AD = Alzheimer disease, AUC = area under curve, MCI = mild cognitive impairment, ROC = receiver-operating characteristic, SE = standard error, SROC = summary receiver operating characteristic.

The inconsistency index values were 0%, 42.1%, 22.9%, 0%, and 0% for sensitivity, specificity LR+, LR−, and DOR, respectively, revealing a possible low-to-moderate heterogeneity between the included studies. The cumulative conversion rate was 42.9% (151/352). For the included studies, the sensitivities were between 83.3% and 100%, the specificities were between 42.1% and 100%, the pooled sensitivity was 94.7% (95% CI: 89.8%–97.7%), the pooled specificity was 57.2% (95% CI: 50.1%–64.2%), the pooled LR+ was 2.08 (95% CI: 1.71–2.55), and the pooled LR− was 0.15 (95% CI: 0.08–0.26) (Figure 1). The pooled diagnostic accuracy was 73.3%, and the random-effects model estimated an overall DOR of 17.66 (95% CI: 8.54–36.51) (*P* = 0.771). The SROC curve showed a Q value of 0.81 ± 0.03 (±SE) and an AUC of 0.88 ± 0.03 (±SE) (Figure 2). The Spearman correlation coefficient was 0.29 (*P* = 0.39), which suggested that there was no threshold effect. Meta-regression to explore potential sources of heterogeneity was not performed because the available data for most covariates were insufficient for conducting this analysis.

#### Short-term Follow-up Subgroup Analysis

**FIGURE 3:**
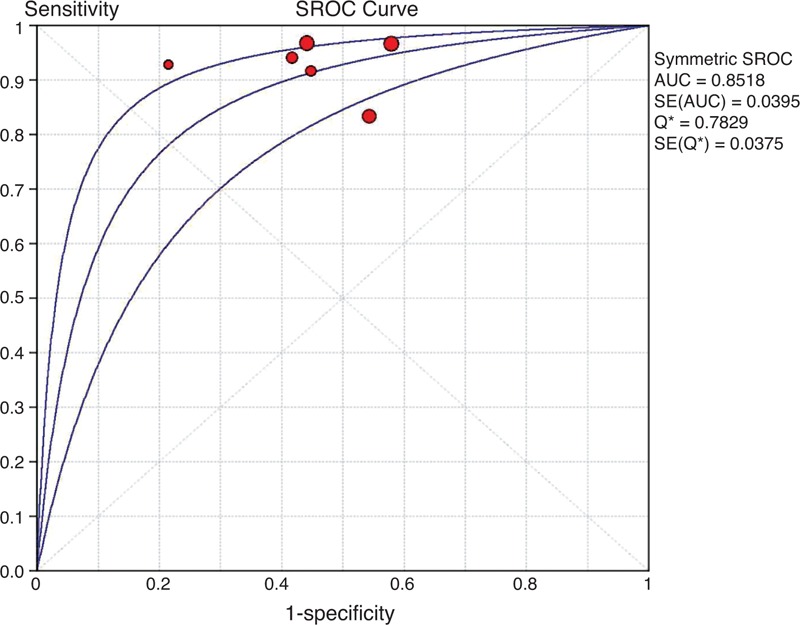
SROC curve of ^11^C-PIB-PET for predicting conversion to AD in patients with MCI in the short-term follow-up subgroup. A weighted symmetric SROC curve with the 95% CI was computed with the Moses-Shapiro-Littenberg method, and the quantitative value of the AUC ± SE was 0.8518 ± 0.0395. We calculated the maximum joint sensitivity and specificity as point Q∗ on a symmetric ROC curve (0.7829 ± 0.0375), which is a global measure of test accuracy. Summary ROC plots display the results of individual studies in ROC space; each study is plotted as a single sensitivity–specificity circular point. The size of points in the plot is proportional to their sample sizes. ^11^C-PIB-PET = ^11^C-Pittsburgh compound B positron emission tomography, 95% CI = 95% confidence interval, AD = Alzheimer's disease, AUC = area under curve, MCI = mild cognitive impairment, ROC = receiver-operating characteristic, SE = standard error, SROC = summary receiver-operating characteristic.

The data extracted from this subgroup had significant homogeneity, with inconsistency index values of 0%, 1.7%, 0%, 0%, and 0% for sensitivity, specificity, LR+, LR−, and DOR, respectively. The cumulative conversion rate was 42.8% (107/250). The sensitivities and specificities of ^11^C-PIB-PET in the prediction of short-term conversion to AD in subjects with MCI ranged from 83.3% to 100% and 42.1% to 83.3%, respectively, and the pooled estimates were 94.4% sensitivity (95% CI: 88.2%–97.9%), 51.0% specificity (95% CI: 42.6%–59.5%) (Supplemental Figure 1, APPENDIX 4, http://links.lww.com/MD/A79), 1.83 LR+ (95% CI: 1.54–2.18) and 0.16 LR− (95% CI: 0.08–0.34). The pooled diagnostic accuracy was 69.6%, and the random-effects model estimated an overall DOR of 13.01 (95% CI: 5.48–30.87) (*P* = 0.48). The SROC curve showed a Q value of 0.81 ± 0.03 (±SE) and an AUC of 0.88 ± 0.03 (±SE) (Figure 3). The Spearman correlation coefficient was −0.086 (*P* = 0.872), indicating that there was no threshold effect.

#### Long-term Follow-up Subgroup Analysis

**FIGURE 4:**
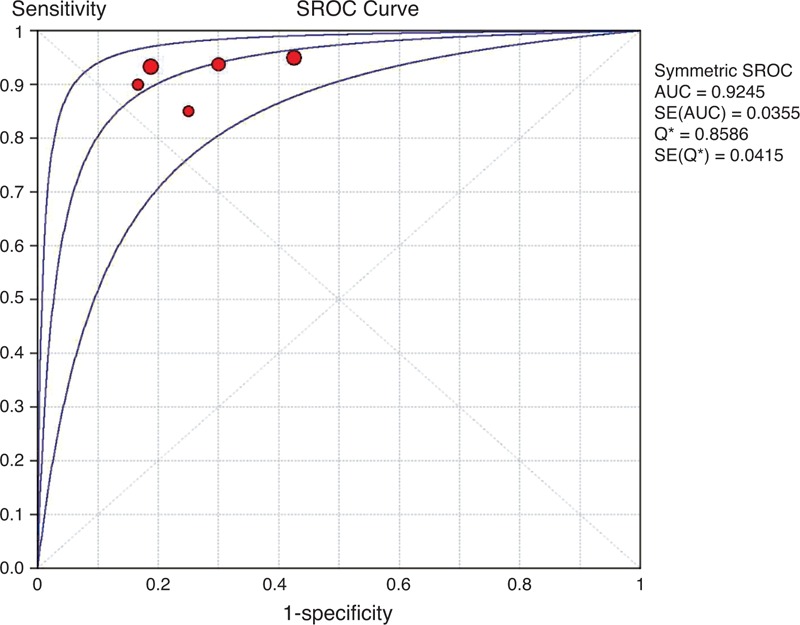
SROC curve of ^11^C-PIB-PET for predicting conversion to AD in patients with MCI in the long-term follow-up subgroup. A weighted symmetric SROC curve with the 95% CI was computed with the Moses-Shapiro-Littenberg method, and the quantitative value of the AUC ± SE was 0.9254 ± 0.0355. We calculated the maximum joint sensitivity and specificity as point Q∗ on a symmetric ROC curve (0.8586 ± 0.0415), which is a global measure of test accuracy. Summary ROC plots display the results of individual studies in ROC space; each study is plotted as a single sensitivity–specificity circular point. The size of points in the plot is proportional to their sample sizes. ^11^C-PIB-PET = ^11^C-Pittsburgh compound B positron emission tomography, 95% CI = 95% confidence interval, AD = Alzheimer disease, AUC = area under curve, MCI = mild cognitive impairment, ROC = receiver operating characteristic, SE = standard error, SROC = summary receiver operating characteristic.

The data extracted from this subgroup had almost no heterogeneity, with inconsistency index values of 0%, 5.6%, 0%, and 0% for sensitivity, specificity, LR+ and LR−, respectively. Moreover, the DOR was also homogeneous across individual studies (*I*^2^ = 0%). The cumulative conversion rate was 43.1% (44/102) in the long-term follow-up subgroup. The overall range of reported sensitivity and specificity was from 88.9% to 100% and 57.9% to 100%, respectively. The pooled estimates for the ^11^C-PIB-PET data were 95.5% sensitivity (95% CI: 84.5%–99.4%), 72.4% specificity (95% CI: 59.1%–83.3.8%), 2.90 LR+ (95% CI: 1.97–4.27), and 0.12 LR− (95% CI: 0.05–0.32). The pooled diagnostic accuracy was 82.4%, and the overall DOR was 36.88 (95% CI: 9.65–140.96) (*P* = 0.981) (Supplemental Figure 2, APPENDIX 5, http://links.lww.com/MD/A79). The area under the SROC curve (±SE) and its Q∗-point (±SE) were 0.93 ± 0.03 and 0.86 ± 0.04, respectively. The summary ROC curve summarizing the accuracy across the 5 studies is shown in Figure 4. The Spearman correlation coefficient was 0.48 (*P* = 0.17), revealing that there was no threshold effect.

#### Comparison of the 2 Different Follow-up Subgroups

Notably, the homogeneity of specificity in each subgroup was significantly higher than that of the included studies (*I*^2^ = 42.1% in all included studies; 1.4% in the short-term subgroup and 5.6% in the long-term subgroup); although metaregression analysis was not performed due to insufficient data, we had sufficient statistical evidence to speculate that the duration of follow-up may be a major cause of the presence of heterogeneity. Additionally, most diagnostic indicators in the long-term follow-up subgroup were superior to those in the short-term follow-up subgroup and the entire group, especially for specificity, LR+, diagnostic accuracy, and DOR, implying that a longer length of follow-up is instrumental in raising the predictive value and diagnostic accuracy of ^11^C-PIB-PET in the early detection of MCI due to AD. In addition, no significant differences were observed between the 2 subgroups in the cumulative conversion rate of MCI (*χ*^2^ = 0.003, *P* = 0.954).

### Sensitivity Analysis

The sensitivity analyses are summarized in Table 7 (APPENDIX 3, http://links.lww.com/MD/A79), which shows that the DORs were significantly altered after removing some studies from each subgroup, suggesting that the diagnosis accuracy was mostly influenced by these studies. We did not observe any obvious impact on our findings.

## DISCUSSION

The recent recommendations from the NIA-AA workgroups on diagnostic guidelines for AD emphasized the importance of appropriate biomarker use in the diagnosis of MCI due to AD. Appropriate biomarker use will provide the highest level of certainty that the subjects with MCI due to AD will progress to AD. Furthermore, biomarkers based on neuroimaging and CSF measures were incorporated into the research criteria for MCI due to AD in these recommendations.^8^ Although subjects who meet the core clinical criteria for MCI and additionally have positive biomarkers for Aβ and neuronal injury will generally progress to AD dementia, some biomarkers have very little diagnostic benefit for predicting conversion, with likelihood ratios suggesting only marginal clinical utility.^26^ Due to potential considerable heterogeneity resulting from different follow-up lengths, a previous study^29^ suggested that ^11^C-PIB-PET scans were not indicated in patients with MCI, except in clinical trials and research studies. The clinical applicability of ^11^C-PIB-PET findings was limited by the heterogeneity of follow-up durations in different studies.

The present systematic review included 11 studies consisting a total of 352 patients with MCI. Overall, the methodological quality of the included studies was moderate to high. Meta-analytically, ^11^C-PIB-PET achieved a very high sensitivity and relatively low specificity in the early diagnosis of AD with MCI. Although low-to-moderate heterogeneity was observed in all included studies, the tests for heterogeneity showed that the included studies in each subgroup were highly homogeneous. In addition, the subgroup analysis indicated that the pooled sensitivity in the long-term follow-up subgroup was slightly superior to that in the short-term follow-up subgroup, whereas the pooled specificity in the long-term follow-up subgroup was far superior to that in the short-term follow-up subgroup. These results indicated that longer follow-up could strengthen the predictive value for MCI due to AD and suggested that standardizing the duration of follow-up may guide the appropriate use of ^11^C-PIB-PET in MCI patients and compel investigators to interpret amyloid imaging in a more reasonable and well-grounded manner.^41^

The duration of follow-up may play an important role in predicting conversion to AD. Although the length of follow-up in the included studies was at least 1 year, the variability in the duration of follow-up was considerable, ranging from approximately 1.0 to 5.2 years. In general, given a longer length of follow-up, a higher proportion of MCI patients at baseline will progress to AD, thus affecting the predictive accuracy of ^11^C-PIB-PET. Similarly, our subgroup analyses also found that studies with a longer follow-up tended to show higher specificities due to the greater number of MCI patients with ^11^C-PIB positivity converting to AD as a function of time; however, we did not find a similar relationship between sensitivity and length of follow-up. Follow-up duration may not significantly impact sensitivity. Although the number of ^11^C-PIB-negative MCI converters may be small (high sensitivity), the clinical outcomes of these MCI patients would not be changed by the length of follow-up. Thus far, the longest follow-up study with 2 ^11^C-PIB-PET scans (at approximately 2 and 5 years) was conducted by Kemppainen et al.^18^ Although this study only included 10 MCI participants, it exhibited the highest specificity (100%) and a higher diagnostic accuracy (90%) of positive ^11^C-PIB-PET scans for predicting MCI to AD conversion at 5 years. Notably, only 1 MCI participant was diagnosed with AD at the time of the scan at 2 years, whereas the others converted between the scan at 2 years and the scan at 5 years. These results differ from the previously reported 42% to 77% specificity of ^11^C-PIB-PET for predicting short-term MCI to AD conversion.^42^ The inconsistent estimates may be explained by the longer follow-up period. In addition, recent studies^3,18,43^ have demonstrated that MCI with ^11^C-PIB negativity at a certain time point did not exclude the possibility of later progression to clinical AD and ^11^C-PIB positivity.

The above finding is consistent with other studies regarding the relationship between Aβ accumulation and other features of cognitive impairment.^3,44^ Aβ accumulation occurs over 2 to 3 decades and can be detected at low levels with ^11^C-PIB-PET at 15 to 20 years before the typical levels found in patients with MCI due to AD are observed. Amyloid deposition is a long-term, dynamic process. The relationship between amyloid accumulation and time is considered to have a sigmoid-shaped trajectory, and when the amyloid load reaches a higher level (standard uptake value ratio >2.7), rates of amyloid accumulation will approach a plateau. MCI is not a direct consequence of amyloid deposition, whereas amyloid deposition is an earlier, upstream pathophysiological event.^45^ Moreover, MCI due to AD conversion of a given amount of amyloid can be modified by many factors. For example, progression may occur sooner in MCI participants with risk-enhancing characteristics, such as other AD pathologies (ie, NFTs), low education, or risk amplification genes. Similarly, cognitive decline may be delayed in MCI participants with risk-reducing characteristics, such as high education or protective genes.^46^ In contrast, these studies suggest that the progression of cognitive status and hippocampal atrophy are not detectable in an individual with MCI due to AD until approximately 5 years before the symptomatic stage of AD.^3,43,44,47^

Cumulative and mean conversion rates of MCI may be another factor associated with the duration of follow-up, and this factor could have also influenced predictive accuracy. The mean conversion rate of MCI to AD ranged from approximately 8.3% to 29.3% per year (approximately 8.3%–24.9% for the short-term and 17.0%–29.3% for the long-term follow-up subgroup) for the included studies in our meta-analysis, which is similar to the previously reported 8.1% to 28.9% per year.^48^ However, due to the lack of available information, the association between mean conversion rates and the length of follow-up was not well elucidated in this review. Whether the mean conversion rates, which are similar to the rates of amyloid accumulation, have an inverted U shape is unknown.^43^ According to recent theoretical models,^3^ longer follow-up durations may yield high cumulative conversion rates in a given timeframe, thus affecting the predictive accuracy of ^11^C-PIB-PET. Although the relationship between the cumulative conversion rate and length of follow-up was not confirmed in our review, some results obtained from the included studies require further attention. For example, the study^18^ with the longest length of follow-up (exceeding 5.2 years) had the highest cumulative conversion rate (90.0%); correspondingly, the studies^39^ with the lowest cumulative conversion rates were in the short-term subgroup. Further studies are needed to confirm this association.

Some other demographic characteristics of MCI may be underlying factors that modify the conversion rate and predictive accuracy of ^11^C-PIB-PET. Amnestic MCI (a-MCI) may be a precursor of AD, whereas nonamnestic MCI (na-MCI) may be a precursor of other types of dementia. Patients with na-MCI are generally believed to have a significantly lower rate of conversion to AD than a-MCI patients. In a longitudinal study with 550 MCI patients,^49^ MCI with storage memory impairment had the highest and closest risk of conversion to dementia, particularly AD. In contrast, another study found lower conversion rates to AD in patients with a single-domain subtype of MCI compared with those with the multiple-domain type, suggesting that the number of impaired cognitive domains rather than the presence of memory impairment predicted the progression to AD. Furthermore, the MCI subtype was diagnostically unstable.^50^ In our review, large variations and unavailable data of MCI subtypes were observed in the included studies; Grimmer et al (2013)^31^ and Wolk et al (2009)^39^ found no clear association between a particular clinical subtype of MCI and ^11^C-PIB-PET positivity. However, subjects with multidomain amnestic symptomatology more frequently exhibited a significant clinical decline compared with individuals with isolated memory impairment in Grimmer et al's study (2013),^31^ which is consistent with a previous study.^50^ None of the included studies analyzed the underlying correlation between ^11^C-PIB-PET imaging, conversion rate, and MCI subtypes; therefore, to clarify the correlation between MCI subtypes and brain Aβ burden, further prospective ^11^C-PIB-PET studies with MCI subtypes should be performed.

Notably, some studies^30,35,39^ in both subgroups generated their own optimal thresholds, prespecified or did not prespecify what constituted a positive or negative test, and some reports^32,35–37,39^ used visual ^11^C-PIB-PET interpretation. This inconsistency is being addressed currently, although the genesis of uniform imaging analysis techniques and thresholds will not alter the diagnostic accuracy of the test; however, uniform imaging analysis will enable easier integration of results across studies.

Some special pathological types of probable AD can also be used to interpret the false-positive and false-negative results, which are described as follows. First, early AD pathology at autopsy has been reported in an ^11^C-PIB-negative patient clinically diagnosed with probable AD.^51^ In addition, a cohort study^52^ indicated that plaques and tangles independently contribute to cognitive impairment in AD pathology without any other primary neuropathologic diagnosis. Furthermore, NFT formation must therefore be either unrelated to amyloid plaque formation, be temporally distinct process, or both.^53^ Some false-negative findings in diagnostic tests may result from these pathological features. Second, some patients with probable MCI by clinical criteria with positive amyloid PET imaging could have had cerebral amyloid angiopathy rather than AD,^54,55^ resulting in false-positive results. In addition, those with probable AD may have had multiple brain pathologies, most commonly AD with macroscopic infarcts, followed by AD with neocortical Lewy body disease. Similar to AD, the pathology underlying MCI may also be heterogeneous.^56^ Currently, it is unknown whether the complicated and mixed pathology influences imaging and diagnosis by ^11^C-PIB-PET; however, we speculate that the mixed neuropathology of probable AD and MCI may play a role in ^11^C-PIB-PET-negative MCI converters. Unfortunately, no study categorized their cohort by postmortem pathological examinations in the longitudinal follow-up.

The present meta-analysis had several limitations. First, not all included studies were of high methodological quality. Although the quality of the studies reported in our analysis was generally moderate to high, the overall methodological and reporting quality of all considered ^11^C-PIB-PET studies was moderate. An international consensus initiative (the STARDdem Initiative) coordinated by the Cochrane Dementia and Cognitive Improvement Group was recently conducted. This initiative aimed to review the current standard of reporting in diagnostic test accuracy studies and cognitive impairment to generate enhanced guidance in addition to the existing reporting guidelines for diagnostic test accuracy studies.^57^ This developing research field supports the acceptance of consistent methodologies and reporting, which would assist future reviews of the diagnostic accuracy of tests and their synthesis in meta-analyses. Second, the included studies did not report sufficient information to assess the effects of baseline MCI characteristics (subtype, sex, age, APOE, and education) on the predictive value of ^11^C-PIB-PET for converting to AD. Significant heterogeneity of specificity was observed between the included studies. In the subgroup analysis, we found probable explanations for the heterogeneity, but other factors that possibly contributed to the heterogeneity remain unidentified. Third, the number of included studies was relatively small, and insufficient information regarding follow-up limited more specific segment analyses in this review. Forth, some included studies have a relatively small sample size, and no large-scale prospective validation of ^11^C-PIB-PET studies with MCI has been conducted, indicating the risk of sampling bias in the included studies.

This is the first such systematic review analyzing the effects of follow-up length on the prognostic value of ^11^C-PIB-PET in this emerging literature. We observed that longer duration studies tended to yield greater accuracy and particularly, specificity. Specifically, the observations made here regarding the utility of ^11^C-PIB-PET in identifying AD prior to the onset of dementia suggest that at the MCI phase, ^11^C-PIB-PET has great utility. Furthermore, long-term follow-up is beneficial for ^11^C-PIB-PET to validate predictive accuracy. Future research may indicate that at even earlier stages (younger individuals without cognitive impairment), Aβ accumulation in the brain will have greater specificity for indicating pathology rather than normal aging. The updated review should determine whether long-term follow-up increases the predictive accuracy of other biomarkers (ie, cerebrospinal fluid Aβ, APOE ε4 allele). Moreover, more uniform approaches to thresholds, imaging analysis, study protocols, and particularly, uniformity follow-up duration, may provide a more homogenous estimate than available here.^26^

Compared with ^18^F-fluoro-2-deoxyglucose PET and structural magnetic resonance imaging, ^11^C-PIB-PET exhibited the best sensitivity but had lower specificity in some predictive accuracy studies with short-term follow-up.^42,58^ Our findings provide convincing evidence that long-term follow-up period could compensate for the lower specificity in longitudinal ^11^C-PIB-PET studies with MCI. Additionally, this observation reflects the predominantly exploratory nature of the use of amyloid imaging to identify disease processes rather than as a diagnostic test with clinical utility. The early detection of MCI due to the AD process is crucial for early therapeutic interventions to delay the onset of the clinical symptoms and slow cognitive decline. By combining our findings with data emerging from other investigators,^3,43^ predicting the timeline for an individual with MCI with a given Aβ burden to reach the levels usually noted in AD may be possible. The development of better, more accessible, and more accurate biomarkers with better specificity is urgently required.
