# Predictive Accuracy of Amyloid Imaging for Progression From Mild Cognitive Impairment to Alzheimer Disease With Different Lengths of Follow-up: A Systematic Review

**DOI:** 10.1097/01.md.0000464207.48049.37

**Published:** 2015-01-16

In the article “Predictive Accuracy of Amyloid Imaging for Progression From Mild Cognitive Impairment to Alzheimer Disease With Different Lengths of Follow-up: A Systematic Review”,^1^ which appeared in Volume 93, Issue 27 of *Medicine*, the title was given incorrectly. The article was a meta-analysis and not a systematic review. The correct title is given below.

Predictive Accuracy of Amyloid Imaging for Progression From Mild Cognitive Impairment to Alzheimer Disease With Different Lengths of Follow-up: A Meta-analysis
